# Non-invasive measurement of a metabolic marker of infant brain function

**DOI:** 10.1038/s41598-017-01394-z

**Published:** 2017-05-02

**Authors:** Maheen F. Siddiqui, Sarah Lloyd-Fox, Pardis Kaynezhad, Ilias Tachtsidis, Mark H. Johnson, Clare E. Elwell

**Affiliations:** 1 Centre for Brain and Cognitive Development, Birkbeck College, University of London, London, WC1E 7HX United Kingdom; 2 Department of Medical Physics and Biomedical Engineering, University College London, London, WC1E 6BT United Kingdom

## Abstract

While near-infrared spectroscopy (NIRS) haemodynamic measures have proven to be vastly useful in investigating human brain development, the haemodynamic response function (HRF) in infants is not yet fully understood. NIRS measurements of the oxidation state of mitochondrial enzyme cytochrome-c-oxidase (oxCCO) have the potential to yield key information about cellular oxygen utilisation and therefore energy metabolism. We used a broadband NIRS system to measure changes in oxCCO, in addition to haemodynamic changes, during functional activation in a group of 33 typically developing infants aged between 4 and 6 months. The responses were recorded over the right temporal lobe while the infants were presented with engaging videos containing social content. A significant increase in oxCCO was found in response to the social stimuli, with maximum increase of 0.238 ± 0.13 μM. These results are the first reported significant change in oxCCO in response to stimulus-evoked activation in human infants and open new vistas for investigating human infant brain function and its energy metabolism.

## Introduction

NIRS is a non-invasive optical imaging technique that uses absorption of near-infrared light, by underlying brain tissue, to quantify changes in concentration of oxygenated haemoglobin Δ[HbO_2_] and deoxygenated haemoglobin Δ[HHb]. These changes provide valuable measures of changes in cerebral oxygenation and haemodynamics. Due to its low cost, portability and usability, NIRS has become an established neurodevelopmental research tool used to study face perception in infancy^1^, language processing^2^ and infant social cognition^3^. It is also being used to investigate atypical development, including studies involving infants at-risk for autism^4^ and children with diagnosed autism^5–7^.

While NIRS haemoglobin based measures are useful markers of brain function, they are also potentially limited given that they only inform on the oxygen delivery component of the neurovascular coupling pathway. Furthermore, changes in concentration of HbO_2_ and HHb may not always arise due to underlying neural activity, but rather may be the result of physiological noise including extracerebral haemodynamics. This can lead to falsely attributing a haemodynamic response to stimulus-evoked brain activity, i.e. false positives. The reader is referred to the work by Kirilina *et al*.^8^ and Tachtsidis & Scholkmann^9^ for a more detailed discussion on the issue of unintentionally measuring haemodynamic responses that are not due to underlying brain activity. NIRS can also provide information about *in-vivo* cellular energy metabolism through measurement of mitochondrial respiratory chain enzyme cytochrome-c-oxidase (CCO). A recent review by Bale *et al*.^10^ provides a detailed discussion of NIRS oxCCO measurements. Located in the inner mitochondrial membrane, CCO is the terminal electron acceptor in the electron transport chain and is responsible for over 95% of oxygen metabolism in the body^11^. The copper A redox centre of CCO has a distinct absorption peak in the NIR spectrum, in its oxidised form^12^. In healthy individuals, the total concentration of CCO *in vivo* remains constant, therefore the NIRS measurement provides a marker of the oxidation status of CCO (oxCCO). A recent animal study by Bainbridge *et al*.^13^ used phosphorus magnetic resonance spectroscopy (^31^P MRS) in parallel with NIRS and found a significant correlation between the ^31^P MRS biomarkers of cerebral energy metabolism and oxCCO. Consequently, oxCCO measurements provide insight into cellular oxygen utilisation and thus oxygen metabolism, thereby providing a potentially more direct and sensitive marker of brain activation than haemoglobin.

While there has been an increasing use of NIRS haemodynamic measures to investigate emerging brain functions in human development, many unanswered questions remain regarding the haemodynamic response function (HRF)^14^ in infants. In particular, the HRF does not always follow a typical profile and can have differing dynamics across age and brain region of investigation^15^, thereby making it challenging to interpret. It has been hypothesised that in the developing brain, these differences may be due to on-going maturation of the neurovascular coupling process itself alongside neuronal development^16^. NIRS oxCCO measurements combined with haemodynamic measures, particularly alongside the use of a mathematical model of cerebral physiology^17, 18^, could provide converging evidence into the mechanisms of typical and atypical haemodynamic responses to neuronal activation.

The aim of this study therefore was to use an in-house developed broadband NIRS system to assess whether oxCCO could be measured in the presence of the haemodynamic response, resulting from cortical neuronal activation, in typical human infants.

## Methods

### Participants

Thirty-three 4-to-6-month-old infants participated in the study (19 females and 14 males; age 159 ± 25 days old). All parents volunteered and gave written, informed consent to take part. Informed consent was also obtained from the parents for publication of any identifying information/images. The study protocol was approved by the Birkbeck Psychology Ethics Committee and all procedures performed were in accordance with the regulations of the Birkbeck Psychology Ethics Committee. The infants were from varied ethnic backgrounds, predominantly White (British/non-British) and therefore had varying skin and hair colour. Neither skin colour nor hair colour was used as exclusion criteria for participants.

### Procedure

**Figure 1:**
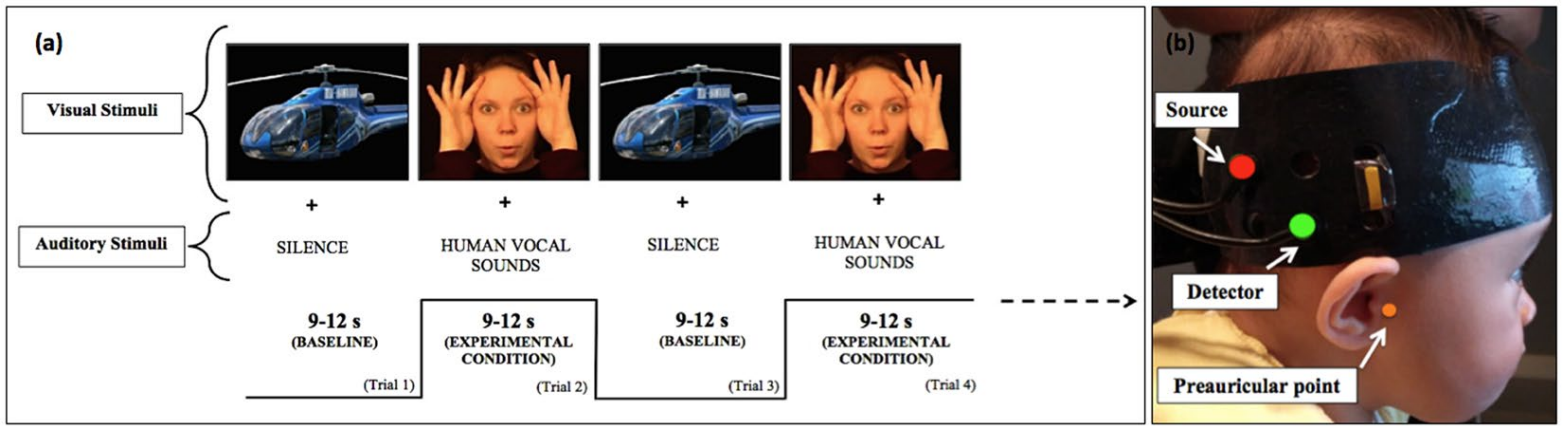
Experimental paradigm and set-up. (**a**) Order of stimulus presentation. (**b**) Image of mini-CYRIL optodes on a participant. A NIRS-MRI age appropriate co-registration map^28^ was used to align the lower front of the array (marked with a yellow stripe) with the right preauricular anatomical landmark. This allowed the source-detector pair to be positioned over the right STS-TPJ, a region previously shown to be activated by viewing dynamic social stimuli^19^.

A 46-in plasma screen was used to display visual and auditory stimuli while the infants were seated on their parent’s lap at a viewing distance of approximately 100 cm. The experimental condition consisted of a range of dynamic social video clips involving biological motion, for example, actors performing “peek-a-boo”. This was accompanied by an auditory component containing human vocal sounds, such as laughter. The baseline condition consisted of static images of different types of transport, for example cars and helicopters, presented randomly for a pseudorandom duration (1–3 s). The displayed experimental and baseline stimuli had equal surface area and subtended an approximate visual angle of 12°. The auditory component of the stimulus was presented at a range between 20–55 dB. The Fig. 1a demonstrates the order of stimulus presentation. The study began with a rest period (20 s minimum) to draw the infant’s attention towards the screen, during which the infant was shown shapes in the four corners of the screen. Following this, the baseline and experimental trials (each 9–12 s long) were alternated for a pseudorandom duration until the infants became bored or fussy. Occasionally, alerting sounds were played during the baseline stimulus to draw the infant’s attention back to the screen. The stimuli used are similar to those described by Lloyd-Fox *et al*.^19^.

### Data acquisition and array placement

NIRS measurements were made using a miniature broadband system which was modified from a larger system (the CYtochrome Research Instrument and appLication system (CYRIL)), developed at University College London^11^. The system, known hereafter as mini-CYRIL, consisted of a miniature Ocean Optics HL2000 white light source using a 20 W halogen-tungsten lamp and an Ocean Optics Ventana VIS-NIR miniature spectrometer^20^. The sampling frequency was 1 Hz. For each infant the attenuation signal was obtained from changes in attenuation of light at 120 wavelengths from 780 to 900 nm^10^.

The mini-CYRIL is a single channel system and the infants wore custom-built NIRS headgear containing one source-detector pair (separation 2.8 cm), placed over the right superior temporal sulcus – temporo-parietal region (STS-TPJ). Figure 1b shows the positioning of the array on an infant.

### Data analysis

Data analysis was carried out in MATLAB (Mathworks, USA). Initial processing involved selection of valid trials by coding for looking time off-line. A trial was considered valid if the infant looked at the screen for a minimum of 4 seconds prior to the onset of the experimental trial and a minimum of 60% of the following experimental trial. To be included in the study, an infant had to have a minimum of 6 valid trials and a typical haemodynamic response must have been exhibited (specified as an increase in Δ[HbO_2_] and either a decrease or no change in Δ[HHb]) in response to the stimulus. Previous studies have reported inverted haemodynamic responses in infants^21–23^, i.e. an increase in Δ[HHb] in response to stimulus rather than in Δ[HbO_2_]. The inverted response is not well understood, and researchers continue to probe the driving factors behind the observed differences in the vascular response during development. Whilst we fully intend to use measures of cytochrome to further these investigations of the mechanisms behind these atypical responses in future work, for this first functional study of cytochrome in infancy we decided to exclude those infants exhibiting an increase in Δ[HHb] and decrease in Δ[HbO_2_] in response to the stimulus. This was done by visual inspection of the data. The wavelet-based motion correction algorithm by Molavi *et al*.^24^ was applied to the attenuation signal of each subject, across all wavelengths, with tuning parameter α = 1.5. The algorithm calculates wavelet coefficients for the NIRS signal using the discrete wavelet transform, which are assumed to have Gaussian distribution that correspond to the physiological signal. The coefficients that are outliers of the Gaussian distribution are identified as artifacts. The tuning parameter α controls the trade-off between the intensity of artifact attenuation and the level of distortion introduced into the NIRS signal. The motion correction algorithm was applied to the data of each subject, to ensure that all data received the same treatment. The tuning parameter α was therefore chosen such as to ensure that the underlying haemodynamic signal was not overly distorted. Following this, the change in attenuation was converted into Δ[HbO_2_], Δ[HHb] and Δ[oxCCO] using the UCLn algorithm^10^. A wavelength-dependent differential path-length factor (DPF) of 5.13 was used^25^. The chromophore concentration changes were filtered using a 5^th^ order Butterworth low pass filter with cut-off frequency 0.225 Hz. The data was then divided into blocks consisting of 4 seconds of baseline prior to onset of the experimental condition, the experimental trial (9–12 s) and the following baseline trial (9–12 s). Linear de-trending was applied to each block of data between the start of the experimental condition and the end of block. The valid blocks for each infant were averaged together and time courses of mean Δ[HbO_2_], Δ[HHb] and Δ[oxCCO] obtained. These were combined to obtain a grand averaged time course of concentration change, across all infants.

To perform statistical comparison of maximum concentration change of each chromophore, in response to the experimental condition versus baseline, a time window between 10 and 18 s post-experimental stimulus onset was selected. This has previously been demonstrated^19^ to be sufficient to include the range of maximum concentration changes, across infants. A one-sample Students t-test was then performed during this window.

### Data availability

The data that support the findings of this study are available on request from the corresponding author M.F.S. The data are not publicly available due to them containing information that could compromise research participant privacy or consent.

## Results

According to the criteria described previously, data from 24 of the 33 infants were included. 3 infants were excluded for failing to look at the minimum number of trials, 1 infant was excluded due to incorrect placement of the array on the infant’s head and 5 infants were excluded as they exhibited an increase in Δ[HHb] and decrease in Δ[HbO_2_] in response to the stimulus, i.e. an inverted response to the experimental stimulus. The grand averaged concentration changes for the 5 infants with inverted responses has been included in the Supplementary information, as Supplementary Figure 1.

**Figure 2:**
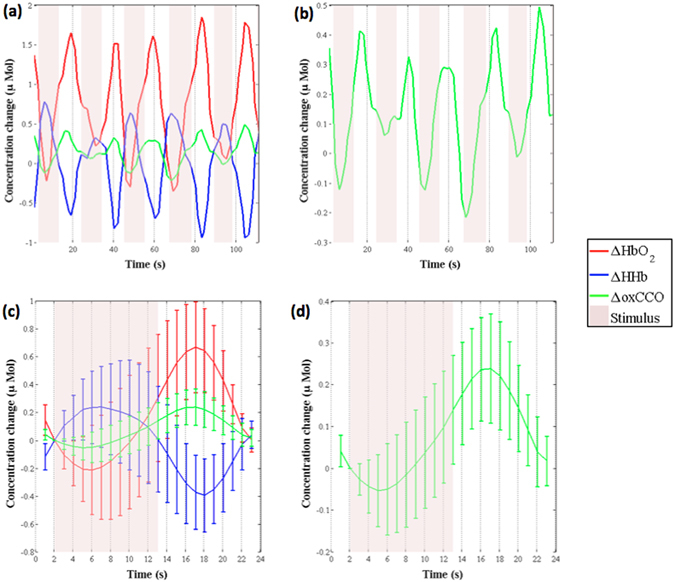
Observed chromophore concentration changes. (**a**) Changes in concentration in HbO_2_, HHb and oxCCO from one participant, across 6 trials, after filtering and applying motion correction. (**b**) Changes in concentration in oxCCO from the same participant, with y-axis re-scaled. (**c**) Grand averaged time course of concentration changes in HbO_2_, HHb and oxCCO, across 24 participants. (**d**) Grand averaged time course of concentration change in oxCCO, with y-axis re-scaled.

**Figure 3:**
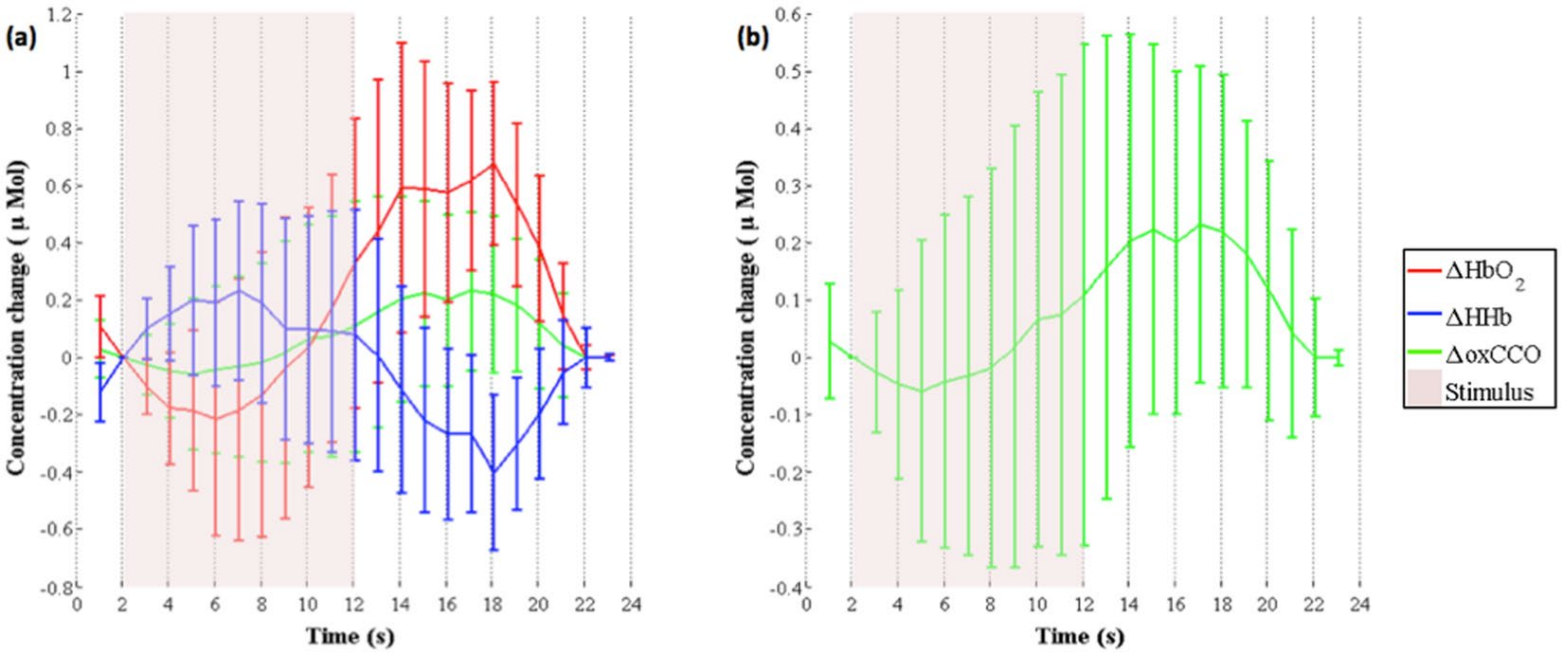
(**a**) Grand median time course of concentration changes in HbO_2_, HHb and oxCCO, across 24 participants. (**b**) Grand median time course of concentration change in oxCCO, with y-axis re-scaled.

Figure 2a displays the changes in concentration of HbO_2_, HHb and oxCCO from a single infant. Figure 2b presents the grand averaged changes in concentrations, for each of the chromophores, across the 24 infants. Figure 3 presents the grand median changes in concentrations across all infants. The one-sample t-test conducted on the group data showed a significant increase from baseline in oxCCO (t_oxCCO_ = 5.710, p_oxCCO_ = 0.000008, t_HbO2_ = 4.387, p_HbO2_ = 0.000174, t_HHb_ = −0.892, p_HHb_ = 0.382, df = 23). An equivalent non-parametric t-test, Wilcoxons signed rank test was additionally conducted on the group data. The results were consistent with the t-test in showing that there was a significant increase in oxCCO in response to the stimulus (z_oxCCO_ = 3.80, p_oxCCO_ = 0.000147, z_HbO2_ = 0.0012, p_HbO2_ = 3.2286, z_HHb_ = −1.2086, p_HHb_ = 0.3037). The maximum change in oxCCO is 0.238 μM. Further Δ[HbO_2_], Δ[HHb] and Δ[oxCCO] from different participants can be found in the Supplementary information.

## Discussion

We have used a novel broadband NIRS system to measure changes in cerebral cellular oxygen metabolism via the measurement of oxCCO in 4-to-6-month-old infants in the presence of typical haemodynamic responses. Our results demonstrate, for the first time in human infants, a significant increase in oxCCO in response to stimulus-evoked activation, and specifically to dynamic social stimuli activating the STS-TPJ region. An earlier study by Zaramella *et al*. investigated brain auditory activation in neonates^26^ and used NIRS to measure Δ[HbO_2_], Δ[HHb] and Δ[oxCCO] over the temporal lobe. While that study reported an increase in HbO_2_ in response to the stimulus, no significant changes were found in the oxCCO signal, most probably due to the limited spectral resolution of the NIRS system used. In contrast, our results clearly demonstrate that by using a broadband NIRS system it is possible to measure changes in oxCCO in infants during functional activation, and hence to obtain critical insights into cellular oxygen utilization. The opportunity to study the physiological processes associated with active neural tissue in this non-invasive, infant friendly way, holds great potential for future research to further our understanding of atypical development in clinical populations. For example, recent studies have suggested a potential link between mitochondrial dysfunction and autism^27^ and measuring oxCCO in a study involving at-risk infants would enable direct investigation of this link. Before this, however, further work needs to be done to characterize the oxCCO signal in typically developing infants, such as through further NIRS infant studies with more complex paradigms and employing the use of a multi-channel broadband system that allows us to measure regional oxCCO brain responses.

## Electronic supplementary material

Supplementary information

## References

[1] Wilcox T, Stubbs JA, Wheeler L, Alexander GM (2013). Infants’ scanning of dynamic faces during the first year. Infant Behav. Dev..

[2] Minagawa-Kawai Y, Mori K, Naoi N, Kojima S (2007). Neural attunement processes in infants during the acquisition of a language-specific phonemic contrast. J. Neurosci..

[3] Grossmann T (2008). Early cortical specialization for face-to-face communication in human infants. Proc. Biol. Sci..

[4] Lloyd-Fox, S. *et al*. Reduced neural sensitivity to social stimuli in infants at risk for autism. *Proc. R. Soc. B Biol. Sci*. **280** (2013). 10.1098/rspb.2012.3026 PMC3619456 23486434

[5] Kita Y (2011). Self-face recognition in children with autism spectrum disorders: A near-infrared spectroscopy study. Brain Dev..

[6] Jung CE, Strother L, Feil-Seifer DJ, Hutsler JJ (2016). Atypical Asymmetry for Processing Human and Robot Faces in Autism Revealed by fNIRS. PLoS One.

[7] Zhu H, Fan Y, Guo H, Huang D, He S (2014). Reduced interhemispheric functional connectivity of children with autism spectrum disorder: evidence from functional near infrared spectroscopy studies. Biomed. Opt. Express.

[8] Kirilina E (2012). The physiological origin of task-evoked systemic artefacts in functional near infrared spectroscopy. Neuroimage.

[9] Tachtsidis, I. & Scholkmann, F. False positives and false negatives in functional NIRS: issues, challenges and the way forward. *J. Biomed. Opt*., doi:10.1117/1.NPh.3.3.030401 (2016). 10.1117/1.NPh.3.3.031405 PMC4791590 27054143

[10] Bale G, Elwell CE, Tachtsidis I (2016). From Jöbsis to the present day: a review of clinical near-infrared spectroscopy measurements of cerebral cytochrome-c-oxidase. J. Biomed. Opt..

[11] Bale G, Mitra S, Meek J, Robertson N, Tachtsidis I (2014). A new broadband near-infrared spectroscopy system for *in-vivo* measurements of cerebral cytochrome-c-oxidase changes in neonatal brain injury. Biomed. Opt. Express.

[12] Jobsis FF (1977). Noninvasive, infrared monitoring of cerebral and myocardial oxygen sufficiency and circulatory parameters. Science (80-)..

[13] Bainbridge A (2014). Brain mitochondrial oxidative metabolism during and after cerebral hypoxia-ischemia studied by simultaneous phosphorus magnetic-resonance and broadband near-infrared spectroscopy. NeuroImage.

[14] Gervain J (2011). Near-infrared spectroscopy: A report from the McDonnell infant methodology consortium. Developmental Cognitive Neuroscience.

[15] Lloyd-Fox S, Blasi A, Elwell CE (2010). Illuminating the developing brain: The past, present and future of functional near infrared spectroscopy. Neurosci. Biobehav. Rev..

[16] Kozberg, M. & Hillman, E. In *Progress in Brain R–esearch* **225**, 213–242 (2016). 10.1016/bs.pbr.2016.02.002 PMC5134842 27130418

[17] Banaji M, Mallet A, Elwell CE, Nicholls P, Cooper CE (2008). A model of brain circulation and metabolism: NIRS signal changes during physiological challenges. PLoS Comput. Biol..

[18] Hapuarachchi, T. *et al*. In *Oxygen Transport to* Tissue XXXVII (eds Elwell, C. E., Leung, T. S. & Harrison, D. K.) 111–120 (Springer New York, 2016), doi:10.1007/978-1-4939-3023-4_14.

[19] Lloyd-Fox S (2009). Social perception in infancy: A near infrared spectroscopy study. Child Dev..

[20] Kaynezhad, P., De Roever, I. & Tachtsidis, I. Optical monitoring of neonatal brain injury: towards the development of compact clinical systems. Electrooptics.com (2016). Available at: http://www.electrooptics.com/news/news_story.php?news_id=2504.

[21] Kozberg MG, Chen BR, DeLeo SE, Bouchard MB, Hillman EMC (2013). Resolving the transition from negative to positive blood oxygen level-dependent responses in the developing brain. Proc. Natl. Acad. Sci..

[22] Zimmermann BB (2012). The Confounding Effect of Systemic Physiology on the Hemodynamic Response in Newborns. Adv. Exp. Med. Biol.

[23] Anderson AW (2001). Neonatal auditory activation detected by functional magnetic resonance imaging. Magn. Reson. Imaging.

[24] Molavi B, Dumont GA (2012). Wavelet-based motion artifact removal for functional near-infrared spectroscopy. Physiol. Meas. Physiol. Meas.

[25] Duncan A (1995). Optical pathlength measurements on adult head, calf and forearm and the head of the newborn infant using phase resolved optical spectroscopy. Phys. Med. Biol..

[26] Zaramella P (2001). Brain auditory activation measured by near-infrared spectroscopy (NIRS) in neonates. Pediatr. Res..

[27] Siddiqui, M. F., Elwell, C. E. & Johnson, M. H. Mitochondrial Dysfunction in Autism Spectrum Disorders. *Autism Open-Access*. 10.4172/2165-7890.1000190 PMC5137782 27928515

[28] Lloyd-Fox S (2014). Coregistering functional near-infrared spectroscopy with underlying cortical areas in infants. Neurophotonics.

