# Supplementary material for: Non-invasive measurement of a metabolic marker of infant brain function
Source: Sci Rep. 2017 May 2;7:1330. doi: 10.1038/s41598-017-01394-z (PMC5430960; doi:10.1038/s41598-017-01394-z)
Supplement: Supplementary file 1 — Supplementary information [file 41598_2017_1394_MOESM1_ESM.pdf]

Non-invasive measurement of a metabolic marker of infant brain function

Maheen F Siddiqui*[1], Sarah Lloyd-Fox[1], Pardis Kaynezhad[2], Ilias Tachtsidis[2], Mark H Johnson[1], Clare E Elwell[2]

[1]Centre for Brain and Cognitive Development, Birkbeck College, University of London, London WC1E 7HX United Kingdom
[2]Department of Medical Physics and Biomedical Engineering, University College London, London WC1E 6BT, United Kingdom

Supplementary Information File

Supplementary Figure 1

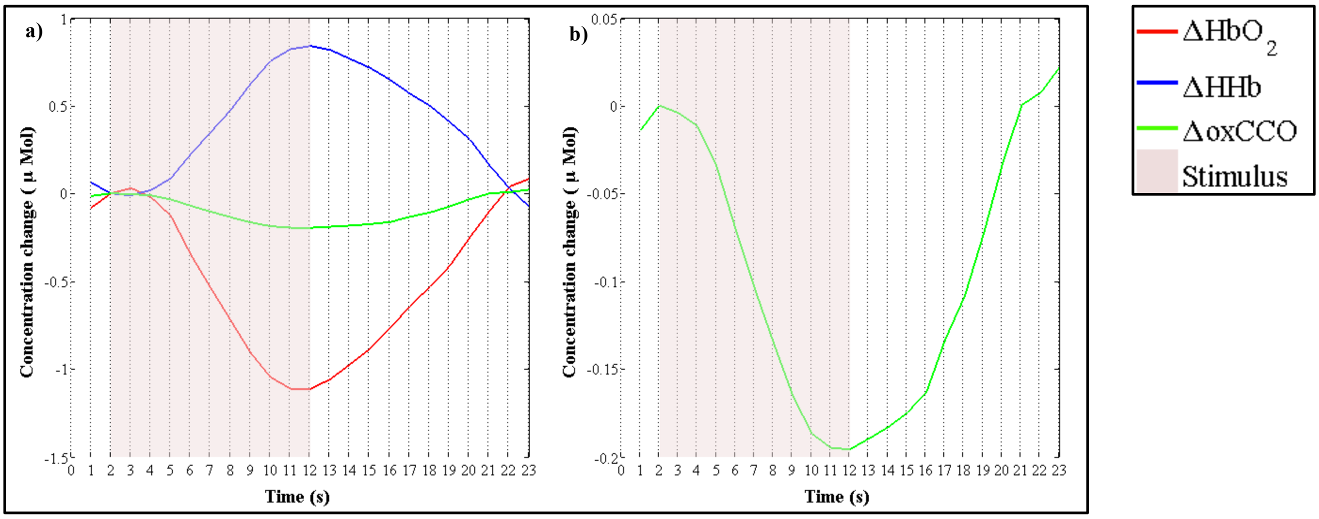

Fig. 1: a) Averaged time course of changes in concentration in HbO_2, HHb and oxCCO across the 5 infants excluded from the study for exhibiting inverted responses to the experimental stimulus. b) Averaged time course of changes in concentration in oxCCO across the same 5 infants, with y-axis rescaled.

Supplementary Figure 2

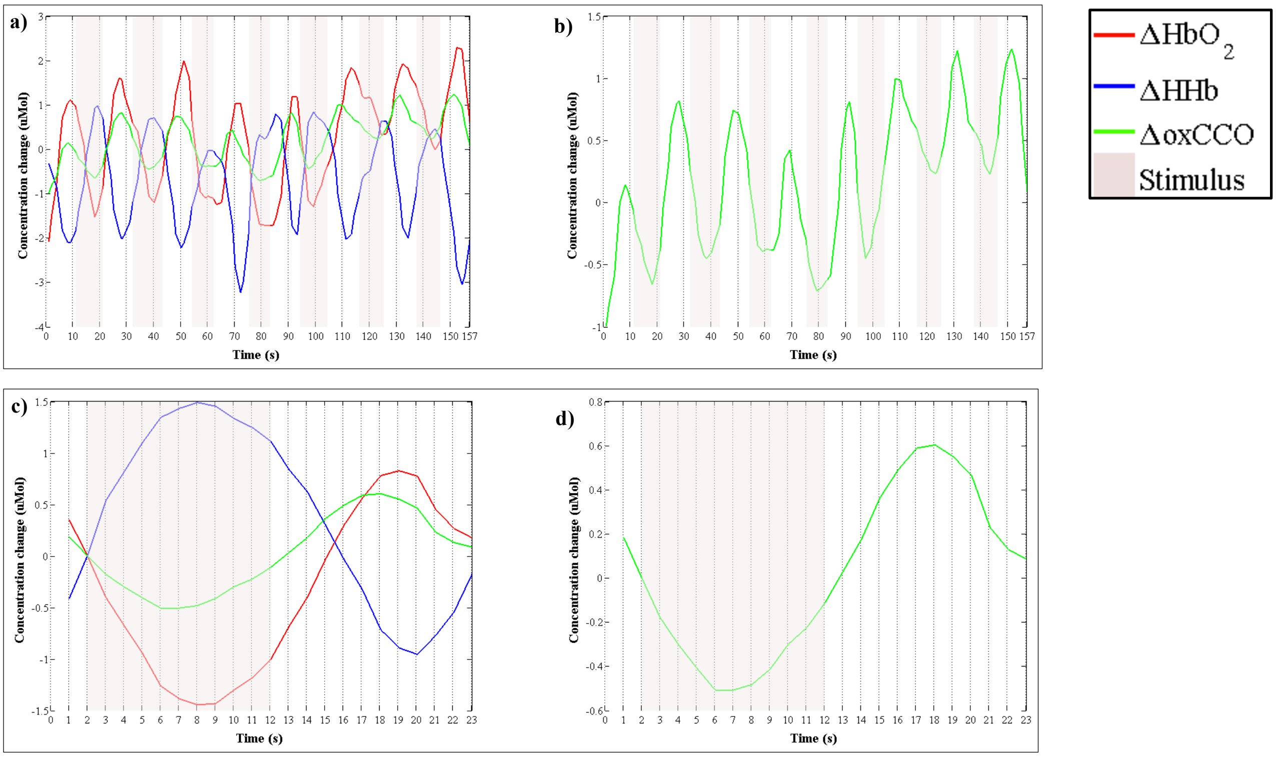

Fig. 2: a) Observed changes in concentration in HbO_2, HHb and oxCCO in Infant 1, across 7 trials, after filtering and applying motion correction. b) Observed changes in concentration in oxCCO in the same infant with y-axis rescaled c) Grand averaged time course of concentration changes in HbO_2, HHb and oxCCO, across all trials in Infant 1. d) Grand averaged time course of concentration changes in oxCCO in the same infant, with y-axis rescaled.

Supplementary Figure 3

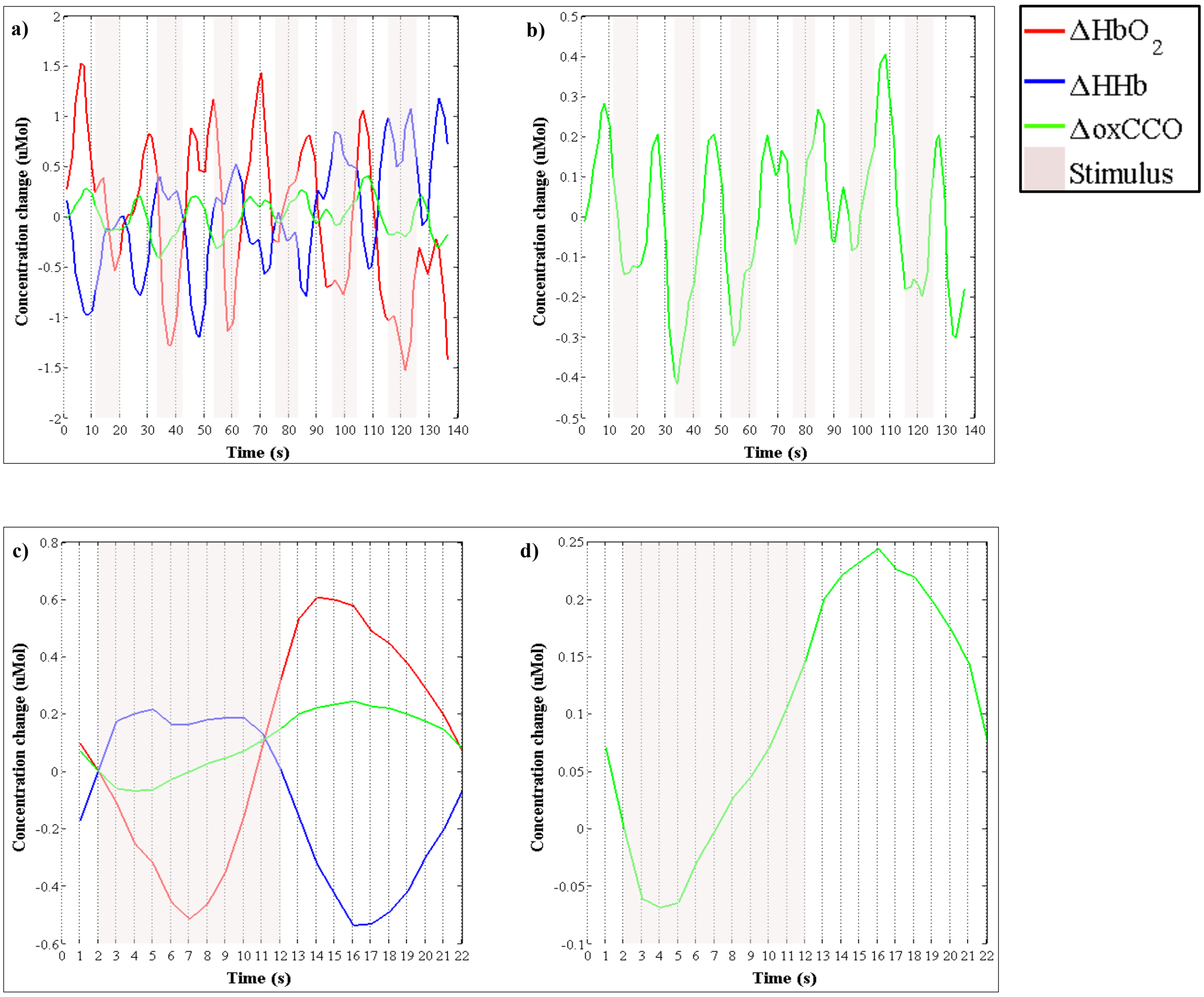

Fig. 3: a) Observed changes in concentration in HbO_2, HHb and oxCCO in Infant 2, across 7 trials, after filtering and applying motion correction. b) Observed changes in concentration in oxCCO in the same infant with y-axis rescaled c) Grand averaged time course of concentration changes in HbO_2, HHb and oxCCO, across all trials in Infant 2. d) Grand averaged time course of concentration changes in oxCCO in the same infant, with y-axis rescaled.

Supplementary Figure 4

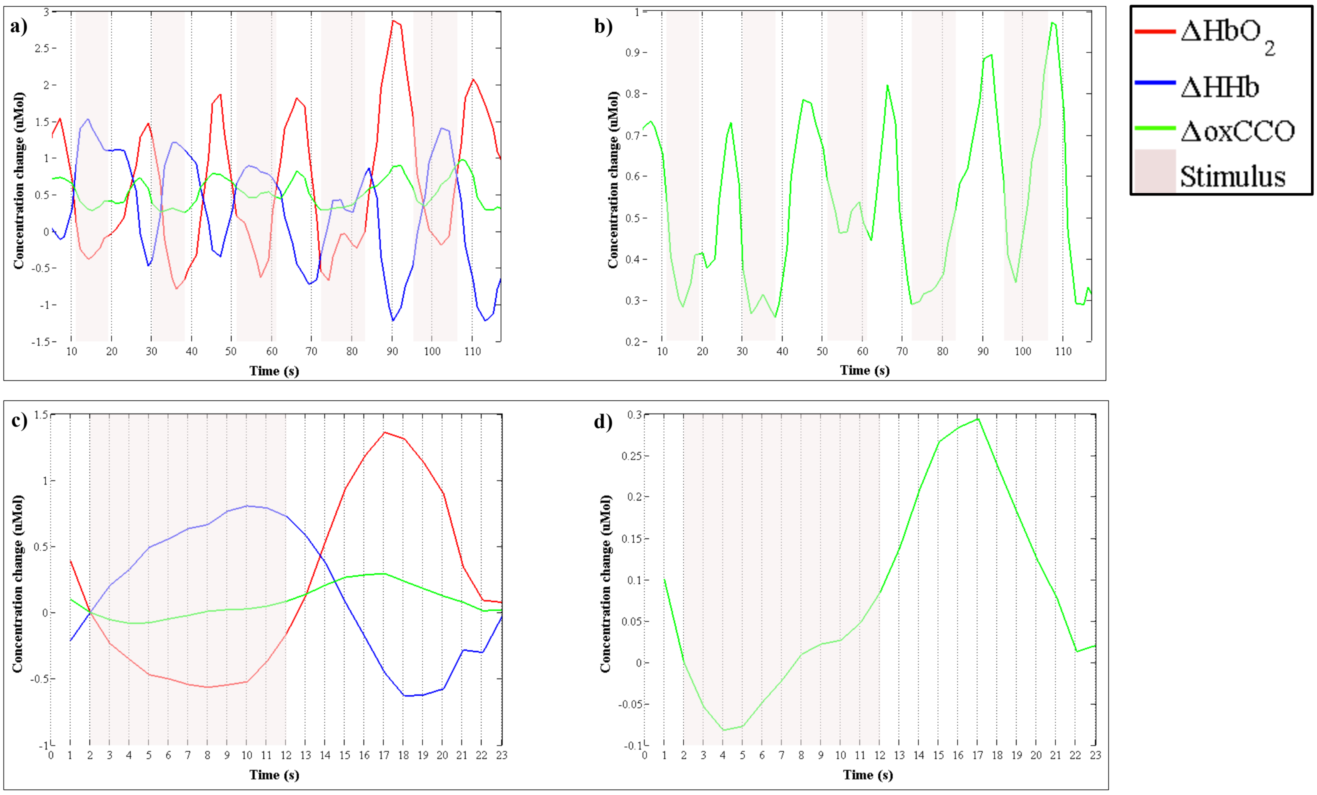

Fig. 4: a) Observed changes in concentration in HbO_2, HHb and oxCCO in Infant 3, across 7 trials, after filtering and applying motion correction. b) Observed changes in concentration in oxCCO in the same infant with y-axis rescaled c) Grand averaged time course of concentration changes in HbO_2, HHb and oxCCO, across all trials in Infant 3. d) Grand averaged time course of concentration changes in oxCCO in the same infant, with y-axis rescaled.

Supplementary Figure 5

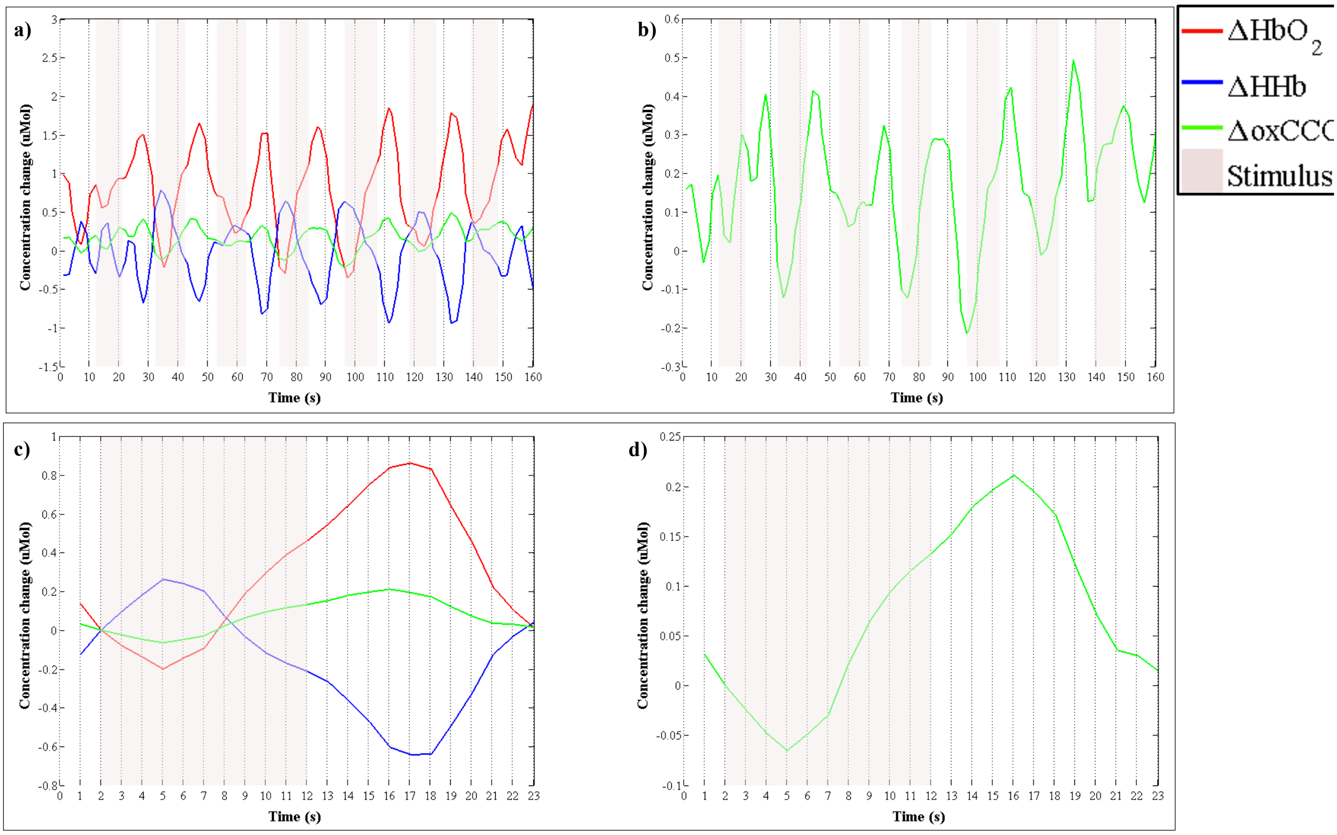

Fig. 5: a) Observed changes in concentration in HbO_2, HHb and oxCCO in Infant 4, across 7 trials, after filtering and applying motion correction. b) Observed changes in concentration in oxCCO in the same infant with y-axis rescaled c) Grand averaged time course of concentration changes in HbO_2, HHb and oxCCO, across all trials in Infant 4. d) Grand averaged time course of concentration changes in oxCCO in the same infant, with y-axis rescaled.

Supplementary Figure 6

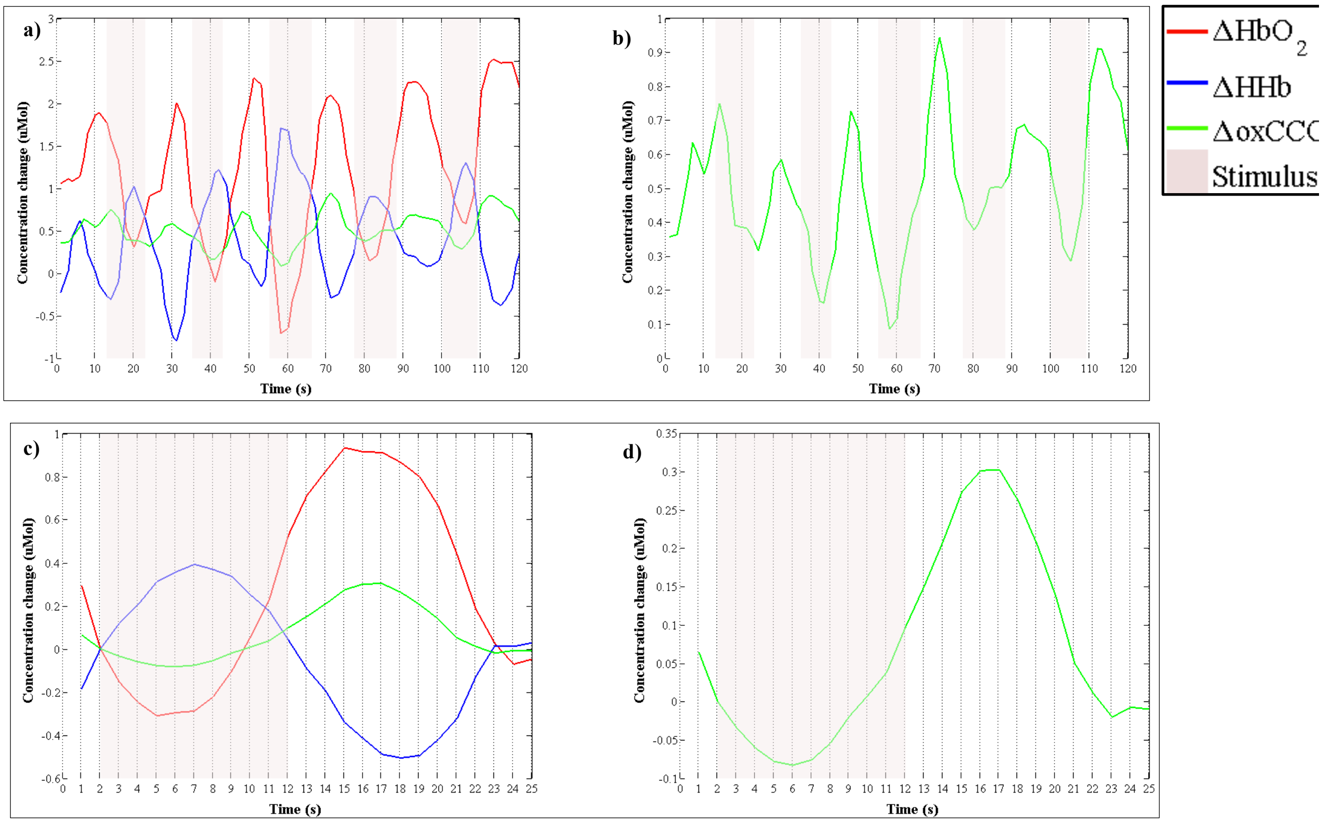

Fig. 6: a) Observed changes in concentration in HbO_2, HHb and oxCCO in Infant 5, across 7 trials, after filtering and applying motion correction. b) Observed changes in concentration in oxCCO in the same infant with y-axis rescaled c) Grand averaged time course of concentration changes in HbO_2, HHb and oxCCO, across all trials in Infant 5. d) Grand averaged time course of concentration changes in oxCCO in the same infant, with y-axis rescaled.

The following figures (supplementary Figures 7 – 14) show the mean concentration change in HbO_2, HHb and oxCCO from all 24 participants.

Supplementary Figure 7

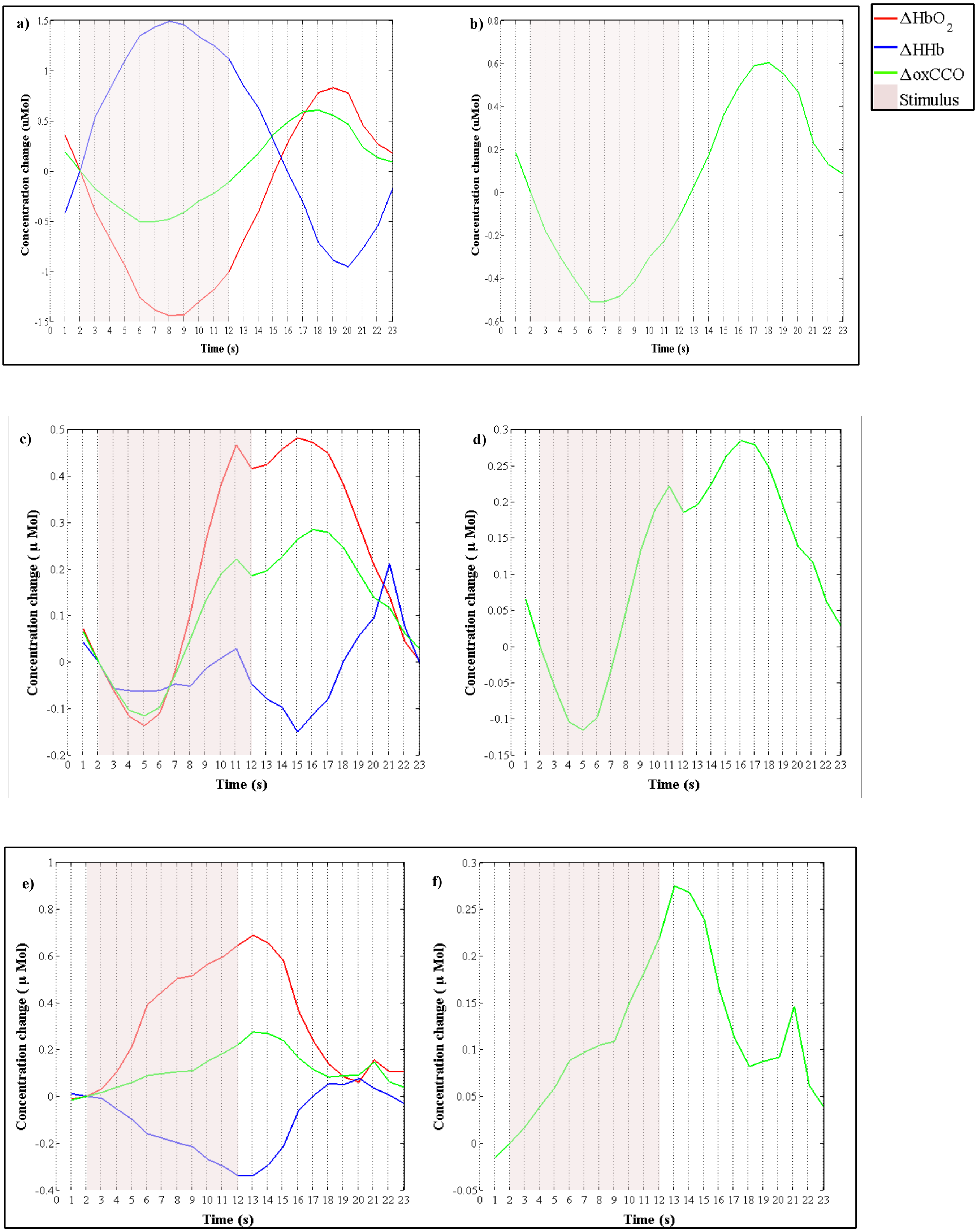

Fig. 7: a) Mean changes in concentration in HbO_2, HHb and oxCCO from Infant 1. b) Mean change in oxCCO in Infant 1. c) Mean changes in concentration in HbO_2, HHb and oxCCO from Infant 2. d) Mean change in oxCCO in Infant 2 rescaled. e) Mean changes in concentration in HbO_2, HHb and oxCCO from Infant 3. f) Mean change in oxCCO in Infant 3, rescaled.

Supplementary Figure 8

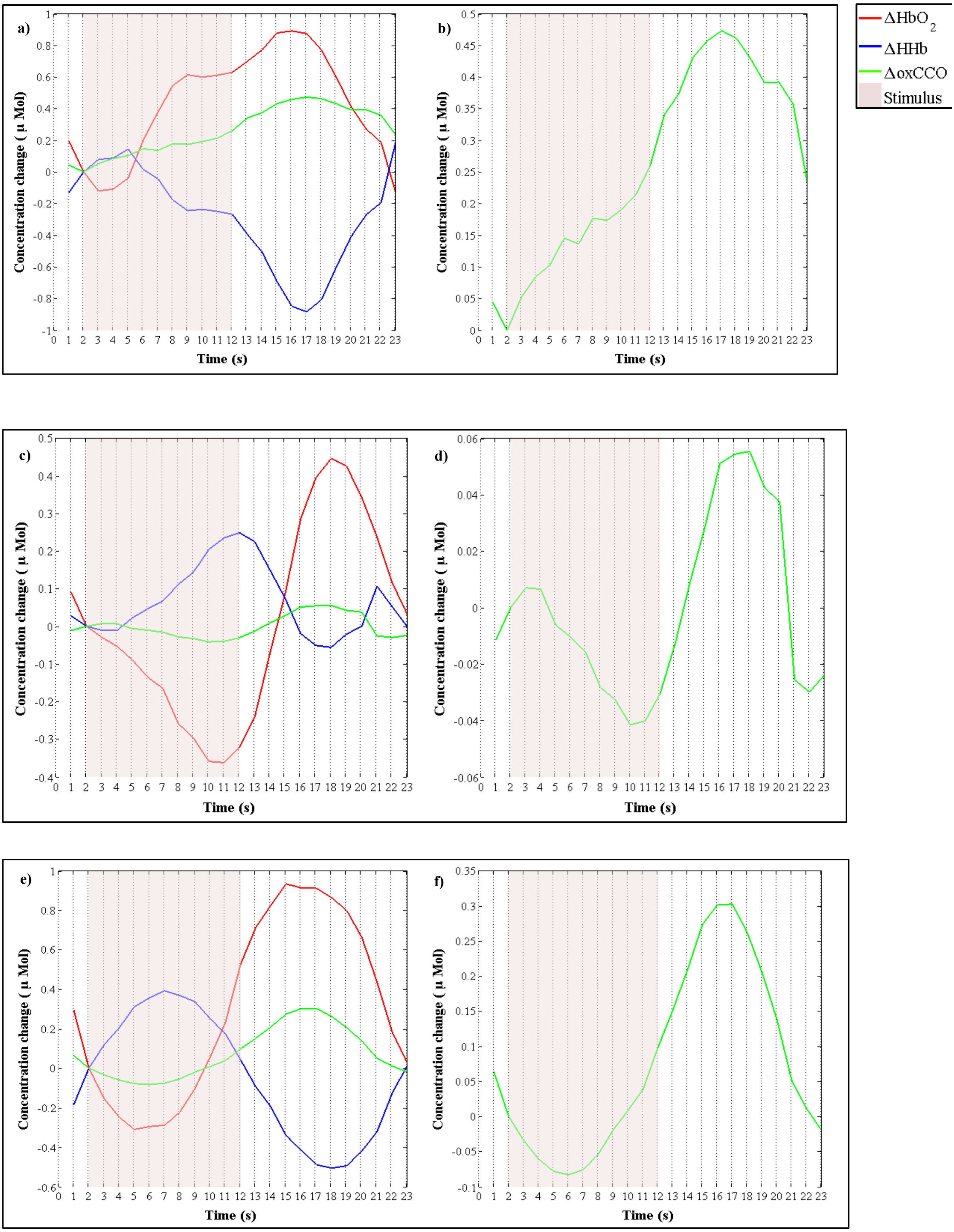

Fig. 8: a) Mean changes in concentration in HbO_2, HHb and oxCCO from Infant 4. b) Mean change in oxCCO in Infant 4, rescaled. c) Mean changes in concentration in HbO_2, HHb and oxCCO from Infant 5. d) Mean change in oxCCO in Infant 5, rescaled. e) Mean changes in concentration in HbO_2, HHb and oxCCO from Infant 6. f) Mean change in oxCCO in Infant 6, rescaled.

Supplementary Figure 9

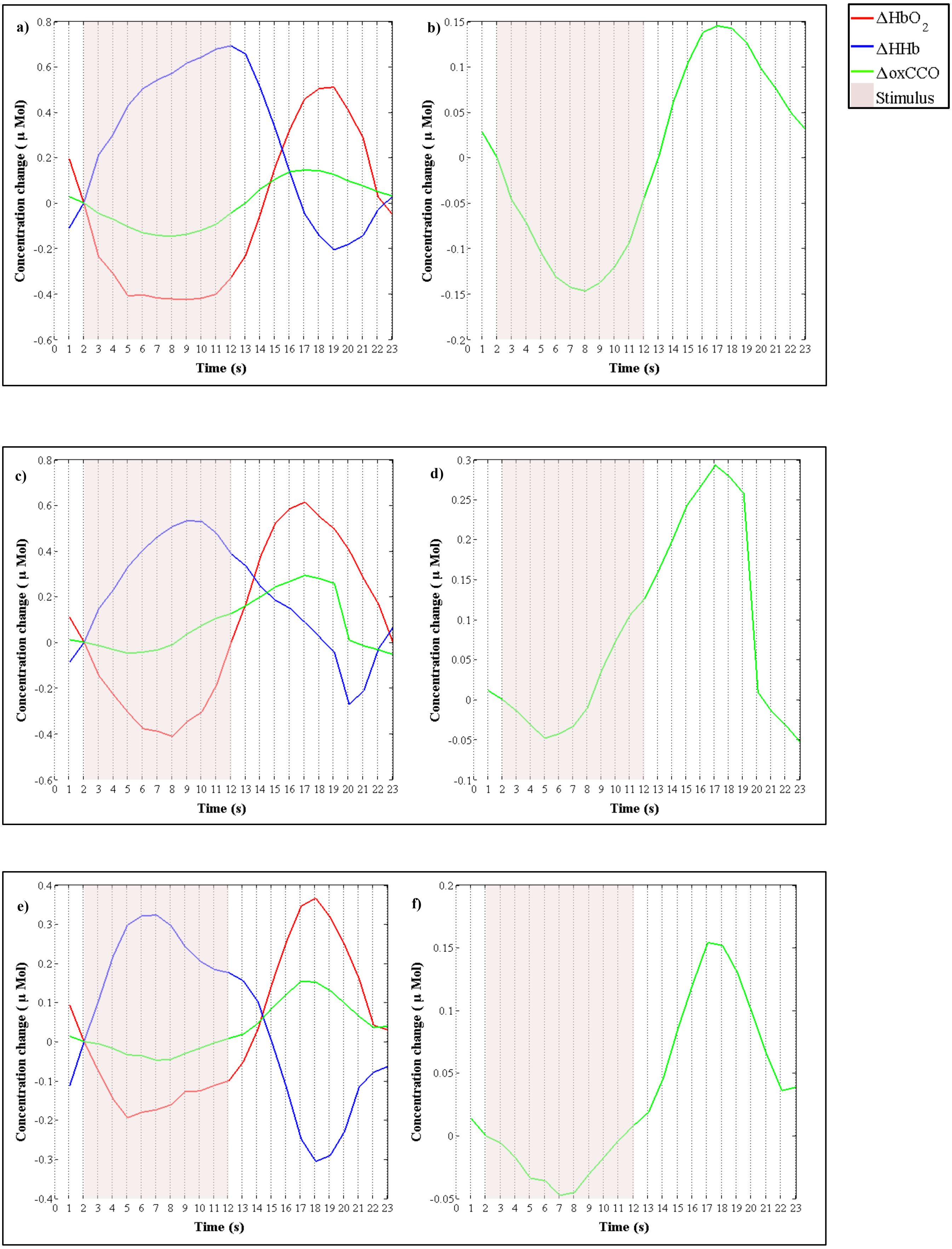

Fig. 9: a) Mean changes in concentration in HbO_2, HHb and oxCCO from Infant 7. b) Mean change in oxCCO in Infant 7, rescaled. c) Mean changes in concentration in HbO_2, HHb and oxCCO from Infant 8. d) Mean change in oxCCO in Infant 8, rescaled. e) Mean changes in concentration in HbO_2, HHb and oxCCO from Infant 9. f) Mean change in oxCCO in Infant 9, rescaled.

Supplementary Figure 10

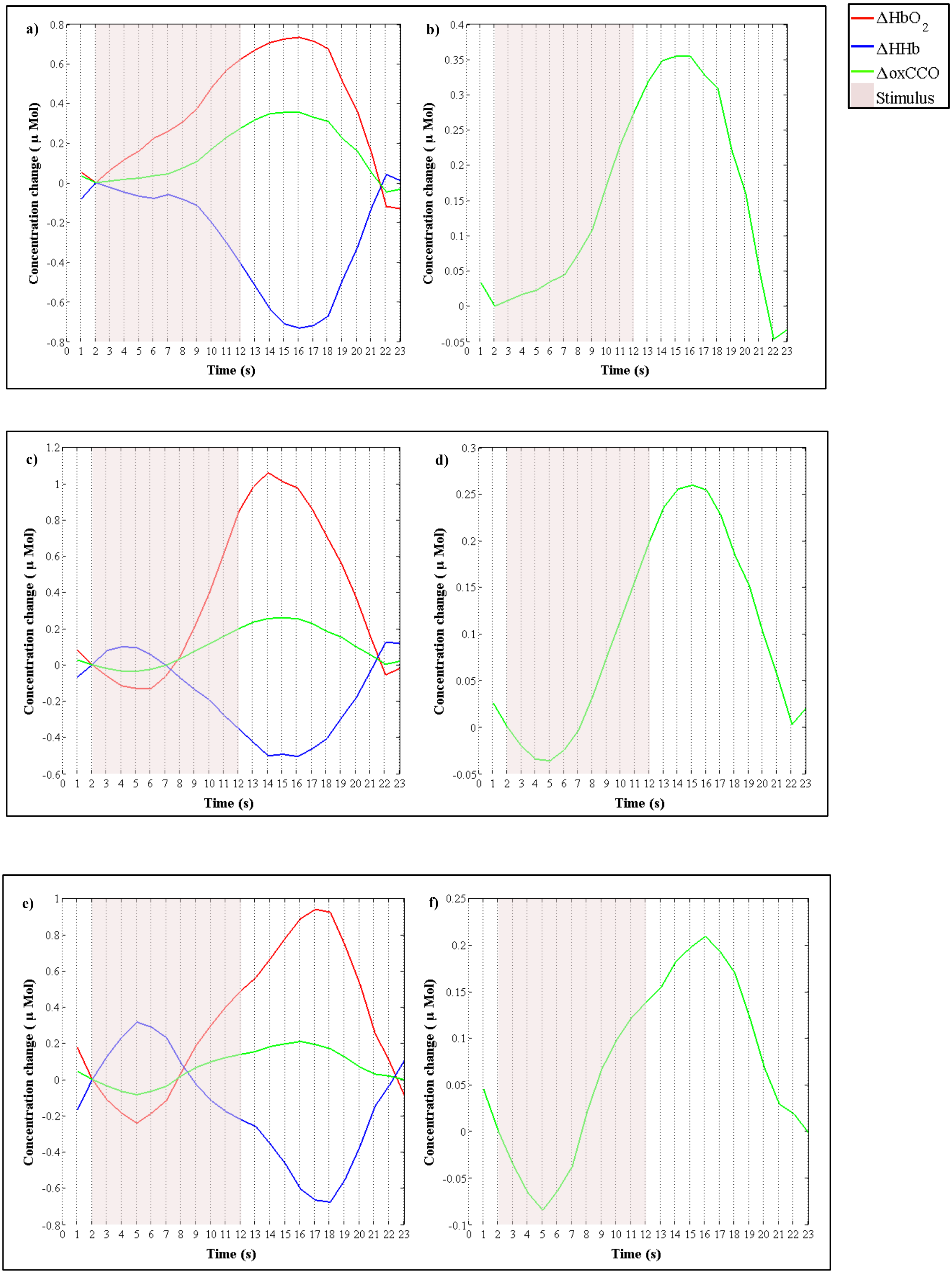

Fig. 10: a) Mean changes in concentration in HbO_2, HHb and oxCCO from Infant 10. b) Mean change in oxCCO in Infant 10, rescaled. c) Mean changes in concentration in HbO_2, HHb and oxCCO from Infant 11. d) Mean change in oxCCO in Infant 11, rescaled. e) Mean changes in concentration in HbO_2, HHb and oxCCO from Infant 12. f) Mean change in oxCCO in infant 12, rescaled.

Supplementary Figure 11

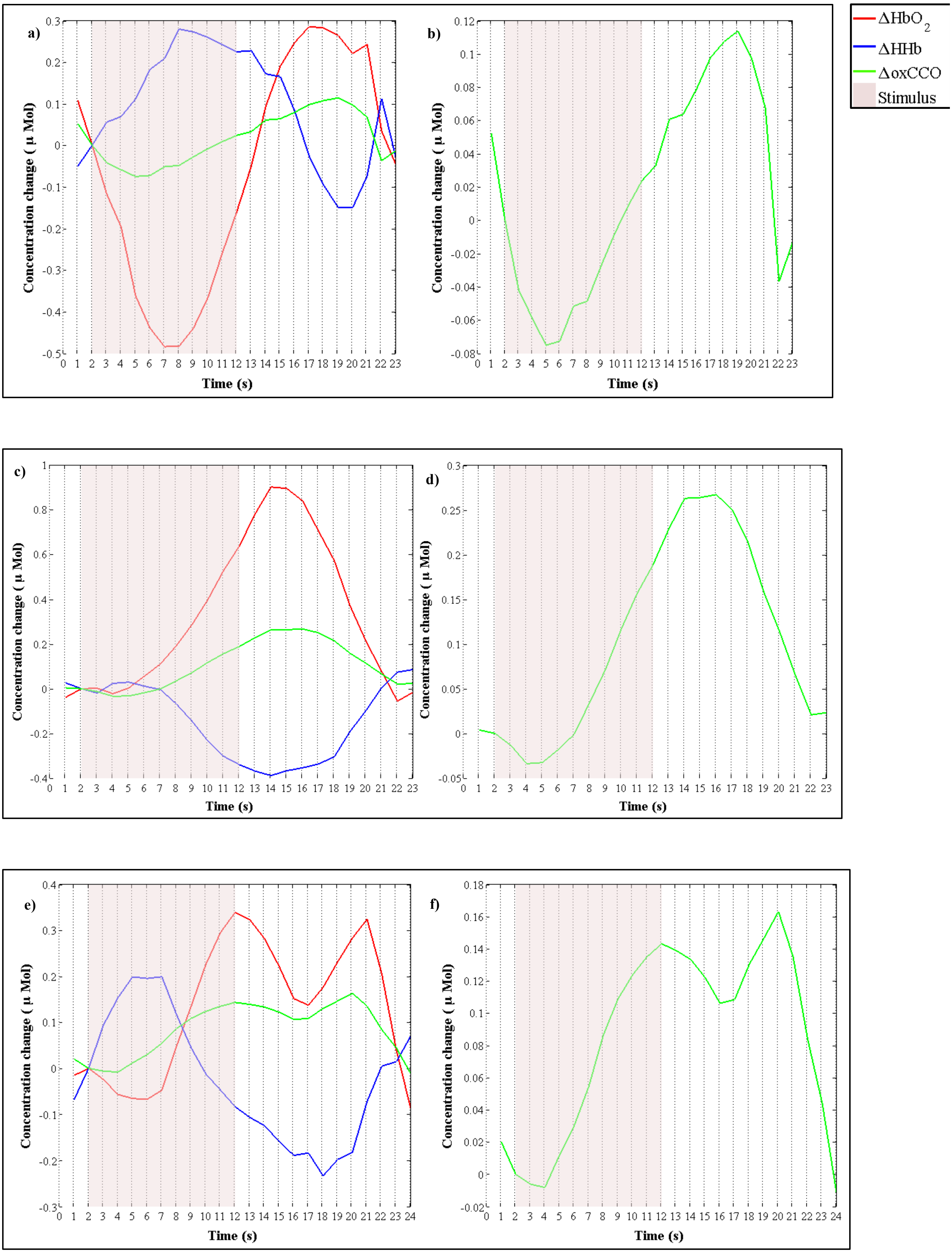

Fig. 11: a) Mean changes in concentration in HbO_2, HHb and oxCCO from Infant 13. b) Mean change in oxCCO in Infant 13, rescaled. c) Mean changes in concentration in HbO_2, HHb and oxCCO from Infant 14. d) Mean change in oxCCO in Infant 14, rescaled. e) Mean changes in concentration in HbO_2, HHb and oxCCO from Infant 15. f) Mean change in oxCCO in infant 15, rescaled.

Supplementary Figure 12

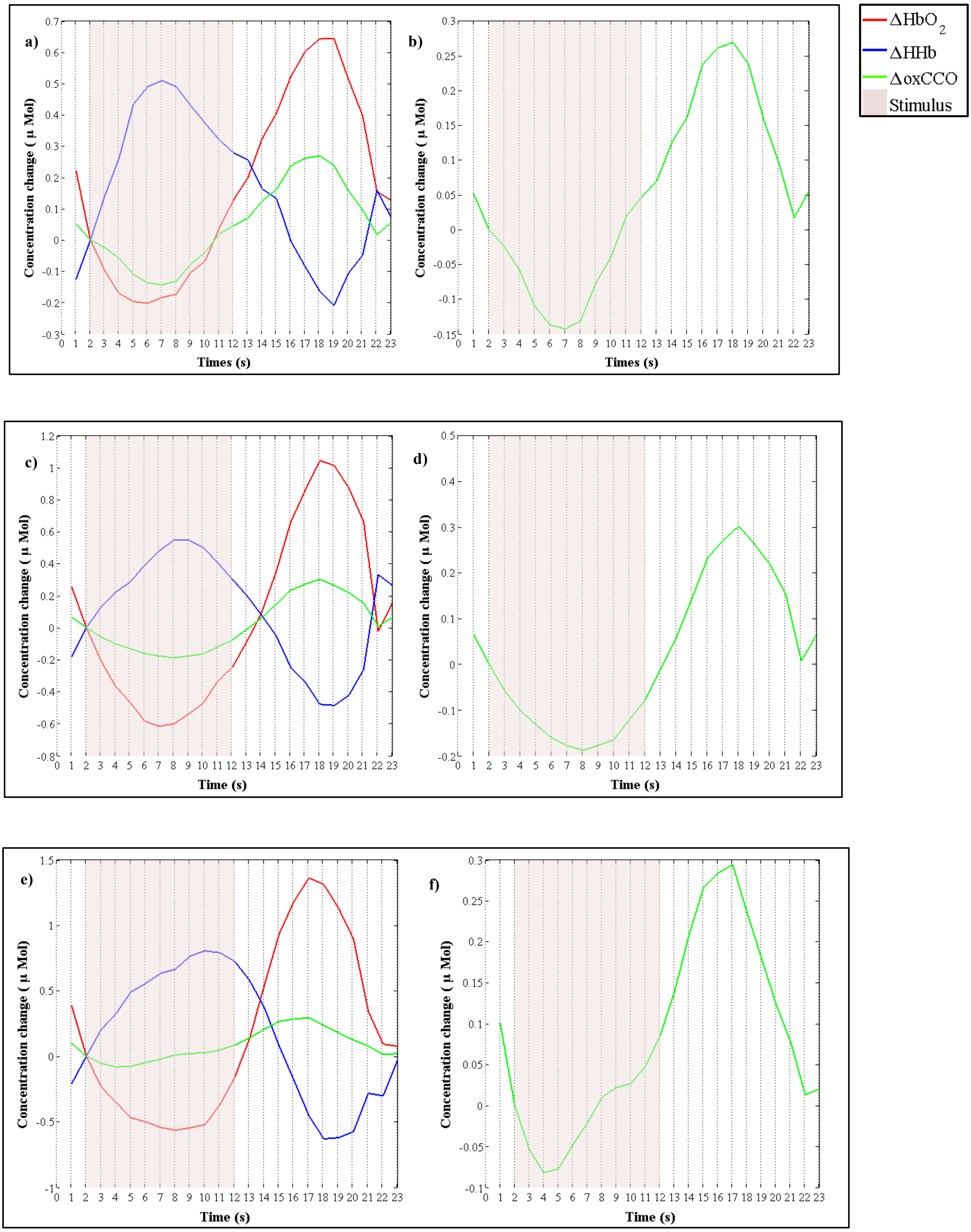

Fig. 12: a) Mean changes in concentration in HbO_2, HHb and oxCCO from Infant 16. b) Mean change in oxCCO in Infant 16, rescaled. c) Mean changes in concentration in HbO_2, HHb and oxCCO from Infant 17. d) Mean change in oxCCO in Infant 17, rescaled. e) Mean changes in concentration in HbO_2, HHb and oxCCO from Infant 18. f) Mean change in oxCCO in Infant 18, rescaled.

Supplementary Figure 13

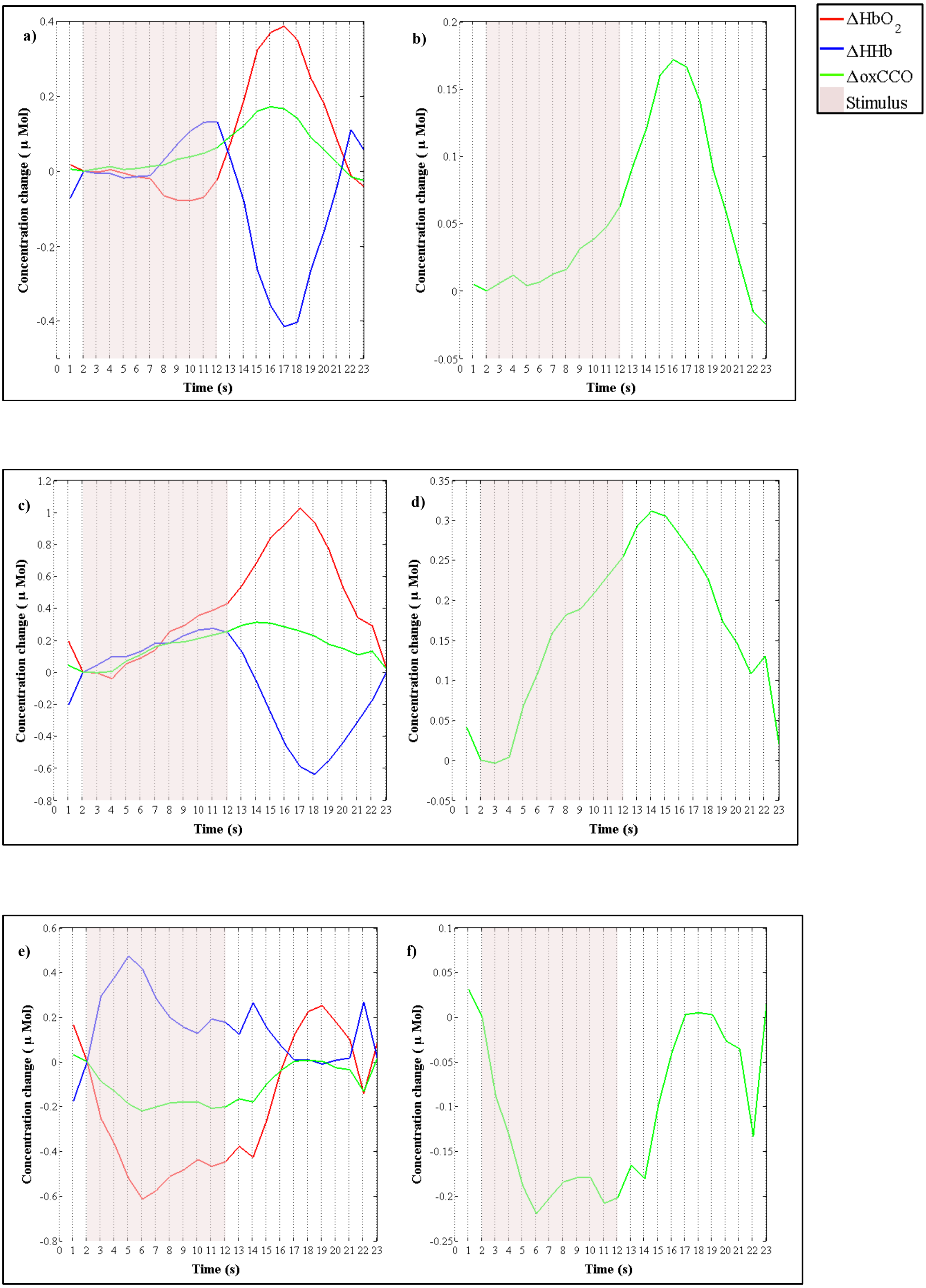

Fig. 13: a) Mean changes in concentration in HbO_2, HHb and oxCCO from Infant 19. b) Mean change in oxCCO in Infant 19, rescaled. c) Mean changes in concentration in HbO_2, HHb and oxCCO from Infant 20. d) Mean change in oxCCO in infant 20, rescaled. e) Mean changes in concentration in HbO_2, HHb and oxCCO from Infant 21. f) Mean change in oxCCO in Infant 21, rescaled.

Supplementary Figure 14

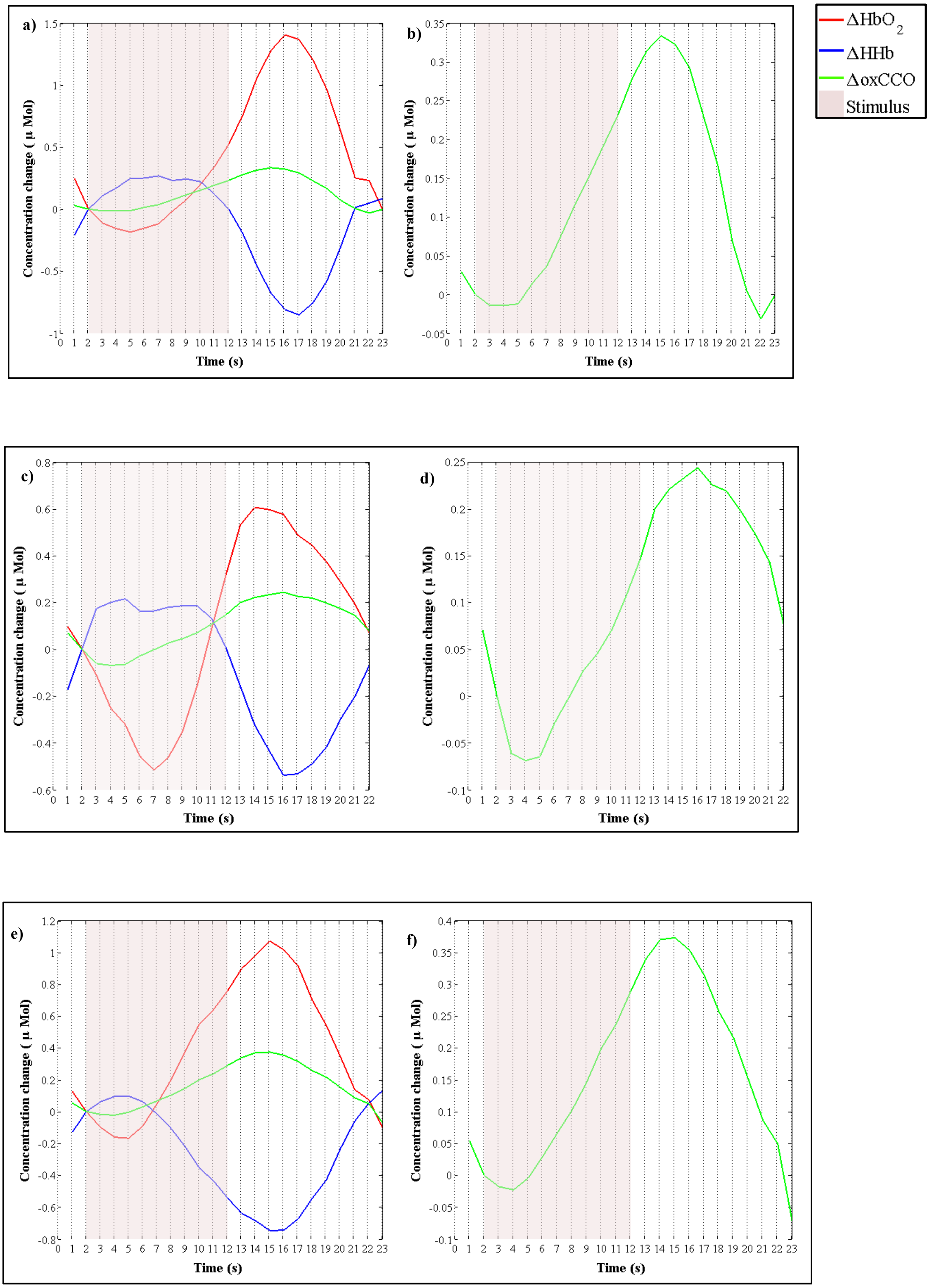

Fig. 14: a) Mean changes in concentration in HbO_2, HHb and oxCCO from Infant 22. b) Mean change in oxCCO in Infant 22, rescaled. c) Mean changes in concentration in HbO_2, HHb and oxCCO from Infant 23. d) Mean change in oxCCO in Infant 23, rescaled. e) Mean changes in concentration in HbO_2, HHb and oxCCO from Infant 24. f) Mean change in oxCCO in Infant 24, rescaled.
